# Supplementary material for: Splice-Junction-Based Mapping of Alternative Isoforms in the Human Proteome
Source: Cell Rep. Author manuscript; Available in PMC 2020 Jan 15. (PMC6961840; doi:10.1016/j.celrep.2019.11.026)
Supplement: 3 [file NIHMS1546469-supplement-3.zip › DF2/PXD006675/LeftCoronaryArtery-27-O95425-SLTQPITTEEVVIAATLQASAHQK.pdf]

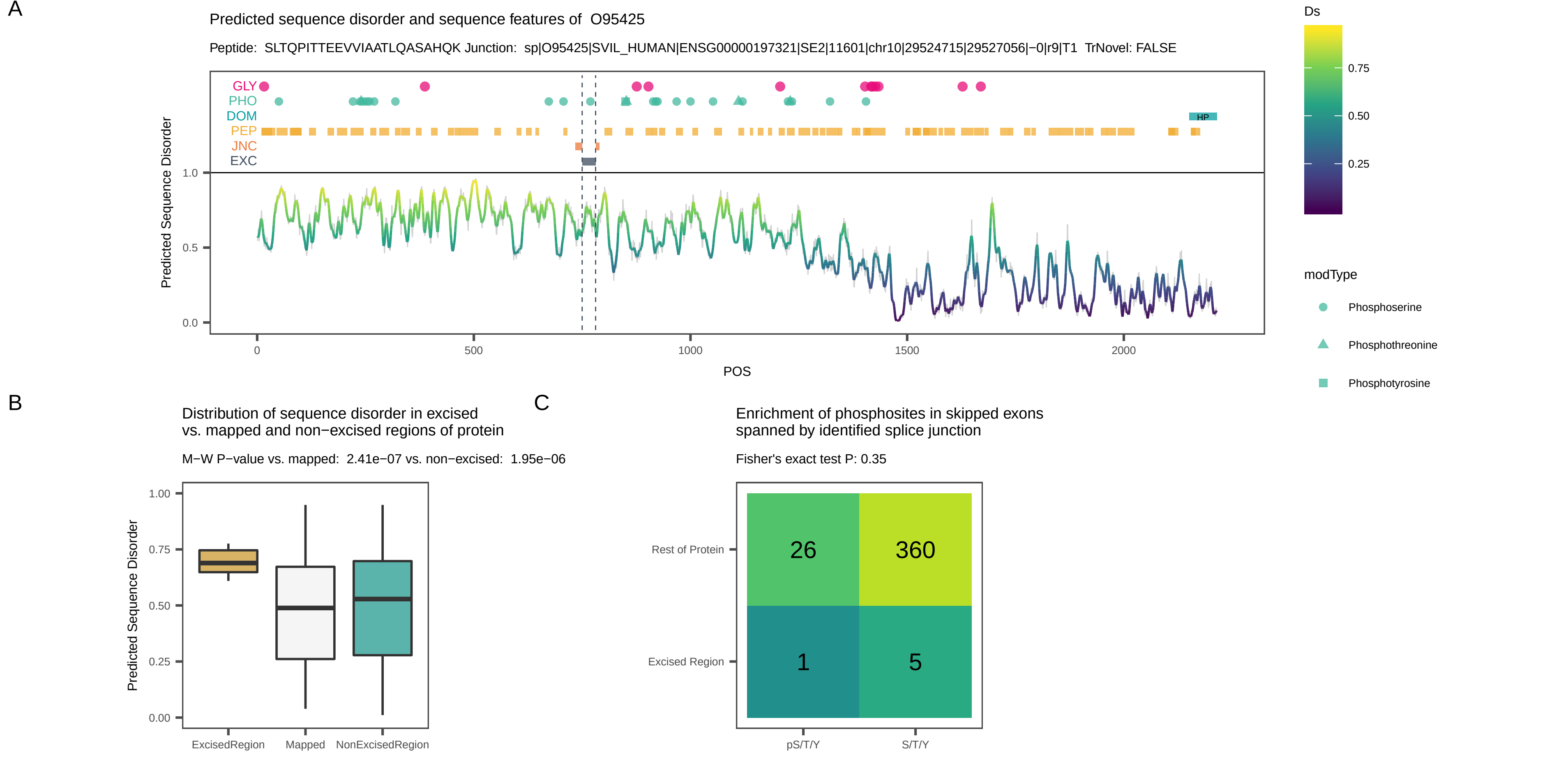
A
Predicted sequence disorder and sequence features of O95425
Peptide: SLTQPITTEEVVIAATLQASAHQK Junction: sp|O95425|SVIL_HUMAN|ENSG00000197321|SE2|11601|chr10|29524715|29527056|−0|r9|T1 TrNovel: FALSE
GLY
PHO
DOM
PEP
JNC
EXC
HP
Predicted Sequence Disorder
1.0
0.5
0.0
0
500
1000
1500
2000
POS
Ds
0.75
0.50
0.25
modType
Phosphoserine
Phosphothreonine
Phosphotyrosine
B
Distribution of sequence disorder in excised vs. mapped and non−excised regions of protein
M−W P−value vs. mapped: 2.41e−07 vs. non−excised: 1.95e−06
Predicted Sequence Disorder
1.00
0.75
0.50
0.25
0.00
ExcisedRegion
Mapped
NonExcisedRegion
C
Enrichment of phosphosites in skipped exons spanned by identified splice junction
Fisher's exact test P: 0.35
Rest of Protein
Excised Region
26
360
1
5
pS/T/Y
S/T/Y
